# Brain Decoding-Classification of Hand Written Digits from fMRI Data Employing Bayesian Networks

**DOI:** 10.3389/fnhum.2016.00351

**Published:** 2016-07-13

**Authors:** Elahe' Yargholi, Gholam-Ali Hossein-Zadeh

**Affiliations:** ^1^School of Electrical and Computer Engineering, University College of Engineering, University of TehranTehran, Iran; ^2^School of Cognitive Science, Institute for Research in Fundamental SciencesTehran, Iran

**Keywords:** Brain decoding-classification, brain connectivity, object representation in the brain, Bayesian network classifiers, similarity analysis

## Abstract

We are frequently exposed to hand written digits 0–9 in today's modern life. Success in decoding-classification of hand written digits helps us understand the corresponding brain mechanisms and processes and assists seriously in designing more efficient brain–computer interfaces. However, all digits belong to the same semantic category and similarity in appearance of hand written digits makes this decoding-classification a challenging problem. In present study, for the first time, augmented naïve Bayes classifier is used for classification of functional Magnetic Resonance Imaging (fMRI) measurements to decode the hand written digits which took advantage of brain connectivity information in decoding-classification. fMRI was recorded from three healthy participants, with an age range of 25–30. Results in different brain lobes (frontal, occipital, parietal, and temporal) show that utilizing connectivity information significantly improves decoding-classification and capability of different brain lobes in decoding-classification of hand written digits were compared to each other. In addition, in each lobe the most contributing areas and brain connectivities were determined and connectivities with short distances between their endpoints were recognized to be more efficient. Moreover, data driven method was applied to investigate the similarity of brain areas in responding to stimuli and this revealed both similarly active areas and active mechanisms during this experiment. Interesting finding was that during the experiment of watching hand written digits, there were some active networks (visual, working memory, motor, and language processing), but the most relevant one to the task was language processing network according to the voxel selection.

## Introduction

The latest advancements in cognitive science point out that distinct activities of the brain might be recognized using neuroimaging data. This attempt for designating brain activities to the recoded brain data is called “brain decoding.”

Brain decoding might be utilized to assist physically handicapped individuals (with motor or speech difficulties) along with enhanced brain–computer interface for brain reading, studying brain activity in non-communicative brain injured patients, and detecting the presence of awareness in these patients (Haynes and Rees, 2006; Boly et al., 2007; Sitaram et al., 2008). Examining the variations in people's perception, learning differences in brain operations such as attention, accessing visual items/materials of dreams, or imaginations along with realizing the brain representation of sensory data are usual purposes of brain decoding (Haynes and Rees, 2006; Thirion et al., 2006; Kay et al., 2008).

Decoding-classification decides which category of stimuli evoked the recorded brain activity. Most decoding-classification studies tried for decoding visual stimulus images of some categories like faces, houses, cats, etc. (Haxby et al., 2001; Carlson et al., 2003; Cox and Savoy, 2003; Hanson et al., 2004; Kamitani and Tang, 2005; O'Toole et al., 2005; Polyn et al., 2005; Ng et al., 2012; Varoquaux et al., 2012). They have applied a restricted variety of stimulus classes with substantial between-class differences and still some decoding performances are certainly not good enough. As a case in point, Eger et al. (2009) performed decoding-classification of computer digits 2, 4, 6, and 8 and obtained a precision of <60%. van Gerven et al. (2010) obtained a discrimination accuracy of more than 70% for decoding-classification of hand written digits 6 and 9. Damarla and Just (2013) achieved a precision of 66% in decoding-classification of computer digit-picture stimuli 1, 3, and 5. Therefore, enhancing decoding-classification continues to be a demanding problem, and it is the target of the current research as well.

In present study, we aimed at decoding classification of hand written digits 0–9. This is a challenging set of stimuli for decoding-classification since all stimuli belong to a single semantic category (Eger et al., 2009) and they are also similar in appearance (similarity exists between some prototypes of the digits like 1 and 7, 5 and 6, 3 and 8, 9 and 8, etc.). For this set of stimuli, it seems that similar looking stimuli will activate same regions/networks of the brain which respond to low level features. Having found an efficient and effective approach for this tough decoding-classification, we might improve decoding-classification generally and employ gained experience in other decoding-classification cases.

Conceptually, brain connectivity is a key feature of each brain state and may be used for brain decoding. A review of brain decoding studies shows that brain connectivity information have been merely utilized as distinguishing features (as inputs for different classifiers, not in determining the classifier itself) in procedures of decoding-classification (Richiardi et al., 2010, 2011; Shirer et al., 2012; Mokhtari and Hossein-Zadeh, 2013). However, several experiments have established the significance of activation correlation between brain locations (Averbeck et al., 2006; Chen et al., 2006). Taking into consideration the brain connectivity, in a recent investigation Yargholi and Hossein-Zadeh (2016) made an effort in hiring brain connectivity information in decoding-reconstruction of two hand written digits 6 and 9. They picked Bayesian networks because these models take advantage of connectivity to spell out probabilistic distributions effectively and provide facilities to exploit probability theory in numerous difficult problems. In this paper, we take the advantages of Bayesian networks for decoding-classification. Additionally, employing the Bayesian network classifiers (other than naïve Bayes classifiers) in brain decoding is a novel application of this tool.

Naïve Bayes is the most basic Bayesian network classifier. This classifier has an assumption of feature independence, far away from some real world circumstances. However, researches in machine learning has found out that more complex Bayesian network classifiers without assumption of independence may result in accuracy increase. So far naive Bayes has been the only Bayesian network classifier used in brain decoding-classification. Following the assumption of feature independence, brain connectivity information are ignored. Therefore, to investigating the effect of brain connectivity on the efficiency of decoding-classification, more complicated Bayesian network classifiers are required. In this way, the enhancement of decoding-classification was studied, exploiting brain connectivity information in the form of dependence between features.

As a way to investigate the accuracy of suggested strategy, whole head functional Magnetic Resonance Imaging (fMRI) was acquired, and decoding-classification was performed on the brain activity (fMRI data) while pictures of hand written digits 0–9 were shown to the subjects.

Another specification of present study is that whole brain fMRI was recorded and the majority of anatomically defined areas from all the lobes were analyzed so there was an opportunity to compare their performance in the same task. Besides, it is possible to compare obtained results with some previous studies which used fMRI data of specific lobes; Eger et al. (2009), and Damarla and Just (2013) used fMRI data of parietal lobe for decoding-classification and van Gerven et al. (2010) performed classification based on fMRI data of occipital lobe. However, in most brain decoding researches, partial brain fMRI data has been employed in the analysis. Even decoding of complex visual stimuli have employed brain activity in early visual regions. Cowen et al. (2014) reported visual stimuli reconstruction based on brain activity outside the occipital lobe. They reconstructed stimuli of face images accurately even when excluding occipital lobe.

In addition to classification, similarity of brain areas in responding to stimuli was also investigated applying data driven exploratory method; hierarchical agglomerative clustering. This similarity analysis unfolded areas responding the same to the stimuli. Another interesting finding of this analysis was determining ongoing mechanism during the experiment. It was fortunate that recoding whole brain images brought us the opportunity of not missing active brain areas or mechanisms.

Next section deals with fMRI data, the theory of Bayesian networks, Bayesian network classifiers, and the procedure of current study for decoding-classification and similarity analysis in details. Section Results contains the results obtained by the proposed method. After that, the Section Discussion and Conclusions presents discussion and conclusions of the results. Besides, some suggestions are brought up to develop brain decoding.

## Methods

The specification of subjects and recorded fMRI data are present in Sections Subjects and MRI data. In order to employ Bayesian network classifiers for decoding-classification, naïve Bayes classifier, and an augmented version of that were selected. In the augmented naïve Bayes classifier, the structure of Bayesian network (edges between features) were extracted using structure learning algorithms and added to the structure of naïve Bayes classifier. In this regard, the Bayesian networks, their parameter and structure learning and Bayesian network classifiers (naïve Bayes classifier and augmented naïve Bayes classifier) are discussed in the following. Then the decoding-classification procedure (including cross validation, voxel selection, and classification) and similarity analysis are explained. Readers familiar with Bayesian networks (BN) may skip Section Bayesian networks since it includes some theories on BNs. While, their application in this research is explained in Section Decoding-Classification.

### Subjects

Three right-handed healthy participants, mean age 28 years (*SD* = 2.65; range = 25–30 years), took part in the study and gave written informed consent approved by Review Board of Tehran University. The participants were not paid for participation.

### Magnetic resonance imaging data

**Figure 1:**
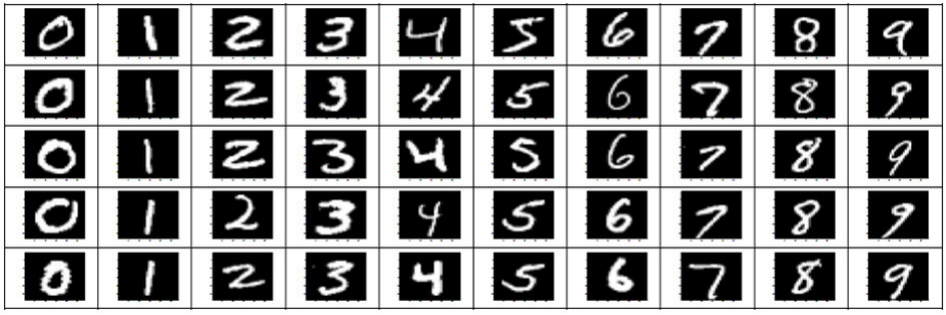
**Examples of stimulus images (hand written digits)**.

The stimuli consisted of 1000 hand written gray-scale digits at a 28 × 28 pixels resolution. For each digit (0–9), 100 unique instances were randomly chosen from the MNIST database (http://yann.lecun.com/exdb/mnist). Figure 1 shows some examples of these stimulus images. The stimuli were presented using Psychtoolbox-3 (Brainard, 1997). The stimulus presentations were scaled and centered to fill the full visual field (9° degrees of visual angle). A central red fixation dot (0.3° of visual angle) was shown during the whole experiments. In each run, after an initial 10 s fixation period, each stimulus was shown for 1 s, followed by 9 s of black background (in each trial, a hand written digit was presented).

The stimuli were presented to subjects through front projection. A screen was made in-house from wood and other non-magnetic materials. During front projection, the projector is mounted in the control room and projects into the scan room through the window, while the screen is floor mounted at the end of the patient bed. The subject lies down on their back inside the scanner and can view front projected images by the use of a mirror inside the scanner.

**Figure 2:**
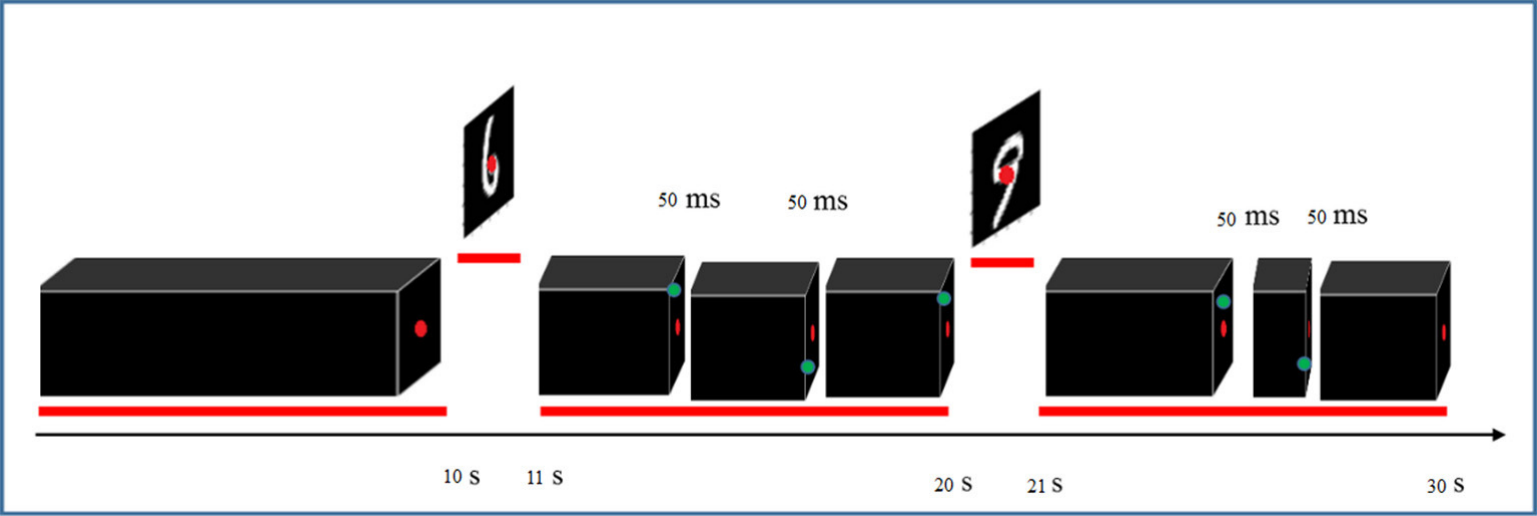
**Paradigm of visual stimulation for recording fMRI data**.

To ensure continuous and complete attention of participants throughout the entire recording sessions, they were asked to focus on red fixation dot at the center of the screen and detect brief (50 ms) appearance of a green dot at random distinct peripheral locations (Figure 2). Detection was reported by pressing a response box button as fast as possible. The green dot appeared three times randomly during each trial with at least 2 s time period between two consecutive appearances. The digits were presented in pseudo-random order where instances of all 10 digits were shown in each 10-consecutive-trial to avoid repetitions of the same digit. The experiment was held in four sessions and each session lasted almost 40 min partitioned into four runs interspersed with 5 min rest periods. Structural scans were also performed in one of the sessions.

Magnetic Resonance Images (MRIs) were collected at the Medical Imaging Center of Imam Khomeini Hospital Complex (Tehran, Iran) with a Siemens 3T MRI system using a 32 channel head coil. Blood oxygenation level dependent (BOLD) functional images were acquired using a single-shot gradient EPI sequence; with repetition time (*TR*) of 2.5 s, echo time (*TE*) of 30 ms, GRAPPA acceleration factor of 4, 83° flip angle, 46 oblique-axial slices, isotropic voxel size 2.2 × 2.2 × 2.2 mm, *FoV* = 222 mm). Moreover, a whole-brain anatomical image was also recorded with the same MR sequence (with the same parameters but more slices) as a whole brain EPI data, to assist the registration. One structural image was also acquired using an MPRAGE sequence (*TR* = 2.3 s, *TE* = 3.03 ms, isotropic voxel size 1 × 1 × 1 mm, 176 sagittal slices, *FoV* = 256 mm). Putting foam cushions around the head during scans, it was attempted to reduce the subject's head motion as much as possible.

fMRI data were analyzed using fMRI Expert Analyzing Tool (FEAT) version 6.00, part of FMRIB Software Library (FSL, version 5.0.7, www.fmrib.ox.ac.uk/fsl). To reach the steady state in the image weights, the first four volumes from the beginning of the data were removed. Then, data were preprocessed including: prewhitening using FILM; motion correction using MCFLIRT (Jenkinson et al., 2002); BET brain extraction; slice timing correction; high pass filtering with a cutoff of 100 s; and no spatial smoothing. Thereafter, a standard general linear model (GLM) was applied to derive the response of voxels to each unique stimulus. There was a set of 16 runs with almost the same duration and GLM was applied to the data of each run independently. The GLM design matrix consisted of regressors each corresponding to one stimulus representation (1 s) and six nuisance regressors encoding movement parameters. Contrast definition also included one contrast for each stimulus to provide us with t-statistic. Then computed response (*t*-value) for each stimulus was used for pattern analysis. The motivation to use *t*-statistic instead of beta is to exclude noisy voxels by incorporating the standard deviation.

To improve the registration, the whole brain EPI volume was used and a three-stage registration was performed using linear registration (FLIRT: Jenkinson and Smith, 2001; Jenkinson et al., 2002); 1. Partial Brain to Full Brain EPI (six degrees of freedom), 2. Full Brain EPI to Structural (seven degrees of freedom), 3. Structural to Standard MNI152-T1-2 mm (twelve degrees of freedom). The resulted transformations were applied to transform EPI volumes from subject space to standard MNI 2 mm space.

Besides, gray-matter masks of frontal, occipital, parietal, and temporal lobe in MNI space were produced using Wake Forest University PickAtlas (http://fmri.wfubmc.edu/cms/software) to parcellate the response volumes and the pattern analysis procedures were applied using responses from only one lobe at a time.

The rest of the analyses were performed using MATLAB (The MathWorks, Natick, MA, USA).

### Bayesian networks

Readers familiar with Bayesian networks (BNs) may skip Section Bayesian networks since it includes some theories on BNs. While, their application in this research is explained in Section Decoding-classification. Bayesian networks are directed acyclic graphs (DAGs) that provide an efficient representation of the joint probability distribution for a set of random variables. Essentially, a Bayesian network consists of two components (*G*, Θ). *G* is a DAG with vertices corresponding to the random variables *X*_1_, …, *X*_*n*_ and edges showing direct dependencies between them. Θ is the set of parameters quantifying the network. Θ includes a parameter θ_*x*_*i*_|Π_*x*_*i*___ = *P*(*x*_*i*_|Π_*x*_*i*__) for each possible value *x*_*i*_ of *X*_*i*_ and Π_*x*_*i*__ of Π_*X*_*i*__ (Π_*X*_*i*__ is the set of parents of *X*_*i*_ in *G*). Parameters are usually shown in tables or functions, one for each variable, in the form of local conditional distributions of a variable given its parents. *G* expresses the independence assumptions: each variable *X*_*i*_ is independent of its non-descendants given its parents in *G*. Based on these independencies, the joint probability distribution is broken down to local conditional distributions (Bishop, 2006; Koller and Friedman, 2009):

(1) $$P(X_{1},\ldots ,\;X_{n})=\prod_{i\;=\;1}^{n}P\left(X_{i}|\Pi_{X_{i}}\right)=\prod_{i\;=\;1}^{n}\theta_{X_{i}|\Pi_{X_{i}}}.$$

Therefore, the number of parameters required to specify a probability distribution is decreased and the posterior probabilities, given evidences are efficiently estimated.

#### Parameter learning in bayesian networks

Having the structure of a BN determined, parameter estimation methods such as Maximum Likelihood Estimation (MLE) or Bayesian Parameter Estimation are applied on dataset to compute the parameters of local conditional distributions, Θ.

Suppose that *D* = {*x*_1_, *x*_2_, …, *x*_*n*_} is a sample set of independent and identically distributed (IID) observations coming from a distribution with an unknown probability density function *f* = *f*(θ) that belongs to a given family of distributions, where θ is a vector of parameters for this family. The goal of MLE is to find θ that predicts *D* well (Koller and Friedman, 2009). Formally, the goal of MLE is to maximize the following likelihood function:

(2) $$L\left(\theta :D\right)=f\left(D|\theta \right)=f\left(x_{1},\ldots ,x_{n}|\theta \right)=\prod_{i\;=\;1}^{n}f(x_{i}|\theta ).$$

In general, likelihood function for *m* nodes *X*_1_, …, *X*_*m*_ of a given BN structure is

(3) $$\begin{array}{l} L\left(\theta :D\right)=\prod_{i\;=\;1}^{n}P(x_{i}:\;\theta )=\prod_{i\;=\;1}^{n}\prod_{j\;=\;1}^{m}P(x_{i_{j}}|y_{i_{j}}:\theta_{j})\\ \;=\prod_{j\;=\;1}^{m}L_{j}(D:\theta_{j}), \end{array}$$

where *x*_*i*_ = (*x*_*i*1_, …, *x*_*mi*_), *x*_*j*_*i*__ is *i*th sample of random variable *X*_*j*_ and *Y*_*j*_ is the parent of *X*_*j*_ (Koller and Friedman, 2009).

#### Structure learning in bayesian networks

The structure of a Bayesian network is either determined by an expert based on his knowledge of the problem or it is extracted by the application of structural learning algorithms on the data set.

Our goal is to impose structure (edges) on naïve Bayesian structure to model the interactions between attributes. There are two different procedures for structure learning: constraint-based and search-and-score (Jordan, 1999). A constraint-based approach starts with a graph with all possible edges. Then, using the available dataset, conditional independencies between variables are investigated to delete some edges. Frequent use of independence tests is the disadvantage of this approach which leads to loss of statistical power. Search-and-score methods rank network structures according to a goodness-of-fit index and perform a heuristic optimization for the best DAG's structures (Murphy, 2004).

Until now, the search-and-score has been mostly preferred to constraint-based approach. The number of DAGs is a super-exponential function of the number of nodes (variables), therefore the full search of DAGs' space is impossible. To tackle with this problem global searches such as Markov Chain Monte Carlo or local searches such as greedy hill climbing are employed (Wesley, 1994).

To extract the structure, in this study, Hill Climbing Greedy Search approach from search-and-score category (Li and Dai, 2005; Russell and Norvig, 2009; Korb and Nicholson, 2010) is used. An empty graph is the start point of the search and the score is computed for all graphs in the neighborhood of the initial graph. Then, the neighbor graph with maximum score is selected for the next step. The neighborhood of a Bayesian network includes graphs that differ only by one insertion, reversion or deletion of an edge from the main graph. This procedure terminates when no better structure is found after a specified number of steps or when the whole structure space is searched.

Friston (2011) defines effective brain connectivity as the influence that one neural system exerts over another, either at a synaptic or population level. Dynamic causal modeling and structural equation modeling are some methods for extracting effective connectivity. These are confirmatory approaches that need a priori models (Kim and Horwitz, 2009; Bajaj et al., 2013, 2014, 2015). In contrast, Zheng and Rajapakse (2006), Rajapakse and Zhou (2007), and Idea et al. (2014) have successfully used structural learning of BNs to discover the effective brain connectivities which is an exploratory approach. This is a remarkable advantage over the above-mentioned conventional methods for extracting effective brain connectivities.

It should be mentioned that the procedure of structure learning of Bayesian Networks for extracting effective connectivity ignores time lags and assumes all effects to be simultaneous (Friston, 2011).

#### Bayesian network classifier

In a Bayesian network classifier, the set of random variables is {*A*_1_, …, *A*_*n*_, *C*}, where the variables *A*_1_, …, *A*_*n*_ are the *attributes* and *C* is the class variable. Consider a graph structure where the class variable is the root (it doesn't have any parents), Π_*C*_ = ∅, and each attribute has a parent set, Π_*A*_*i*__ for 1 ≤ *i* ≤ *n*. For this graph structure, Equation (1) yields

(4) $$P(A_{1},\ldots ,\;A_{n},C)=P(C)\prod_{i\;=\;1}^{n}\;P\left(A_{i}|\Pi_{A_{i}}\right)$$

and applying the definition of conditional probability,

(5) $$P(C|A_{1},\ldots ,\;A_{n})=\alpha .P(C)\prod_{i\;=\;1}^{n}\;P\left(A_{i}|\Pi_{A_{i}}\right)$$

is derived, where α is a normalization constant (Friedman et al., 1997).

### Naïve bayesian classifier

**Figure 3:**
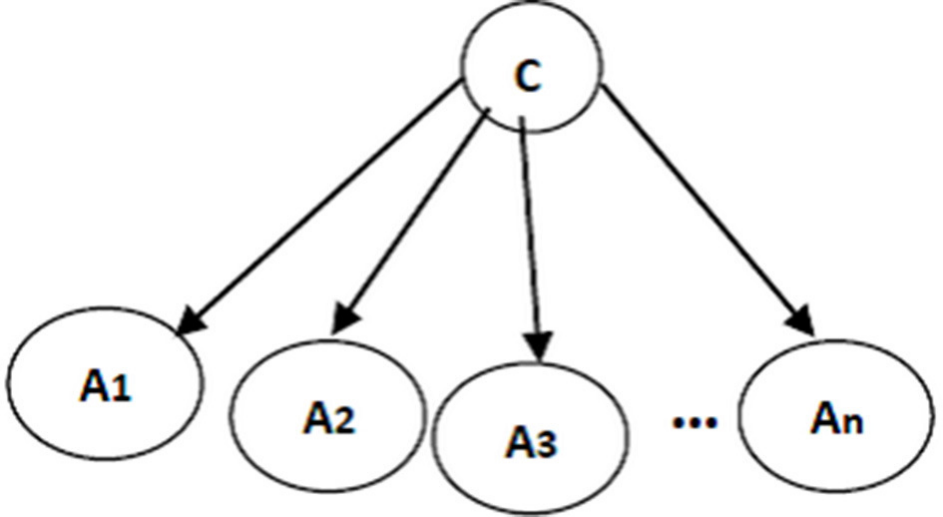
**A naïve Bayes classifier; *A*_1_, …, *A*_*n*_ are attribute nodes, C is the class label node and there are edges between class label node and each attribute node**.

The structure of a naïve Bayes classifier, a simple Bayesian network classifier, is shown in Figure 3. This network structure clearly bears the assumption of a naïve Bayesian classifier; every attribute *A*_*i*_ is independent from other attributes, given the state of the class variable since the local conditional probabilities include just an attribute and the class variable:

(6) $$\begin{array}{l} P(C|A_{1},\ldots ,\;A_{n})=\alpha .P(C)\prod_{i\;=\;1}^{n}\;P\left(A_{i}|\Pi_{A_{i}}\right)\\ \;=\alpha .P(C)\prod_{i\;=\;1}^{n}\;P\left(A_{i}|C\right). \end{array}$$

The conditional probability of each attribute *A*_*i*_ given the class label *C* is learned from the data. Then, classification is performed using Bayes rules to estimate the probability of each state for class variable, given specific values for *A*_1_, …, *A*_*n*_. Finally, the class with the highest posterior probability is chosen as the classifier prediction.

Naïve Bayes classifiers have yielded acceptable results in different classification tasks. However, its assumption on attribute independences is unrealistic and it should be noted that, if two attributes are correlated, then the naive Bayesian classifier may over-amplify the weight of the evidence of these two attributes on the class variable just due to the assumption of attribute independences (Friedman et al., 1997).

### Augmented naïve bayes classifier

**Figure 4:**
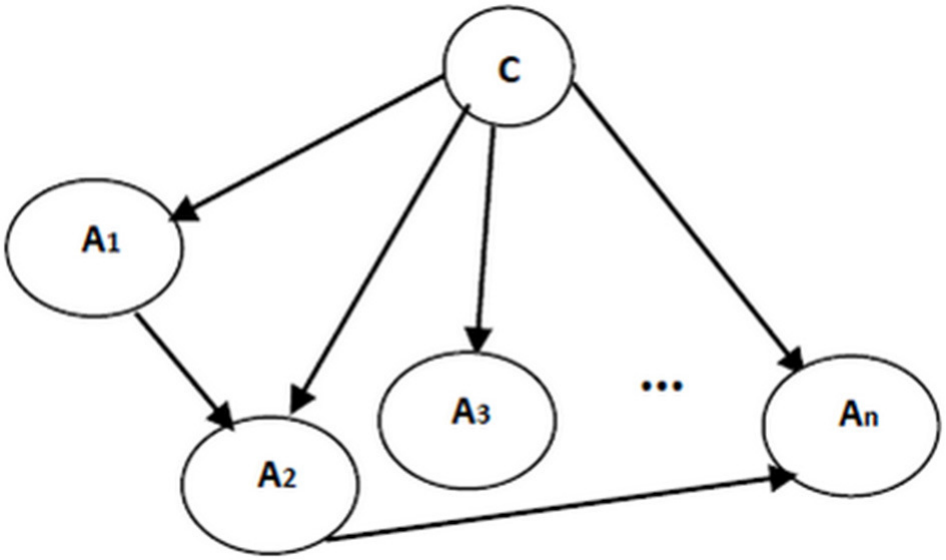
**An augmented naïve Bayes classifier; *A*_1_, …, *A*_*n*_ are attribute nodes, C is the class label node and there are edges between attribute nodes plus edges between class label node and each attribute node**.

Naïve Bayes classifier's assumption on the attribute independences is undoubtedly unrealistic. To effectively address this limitation and to improve the performance of the classification, the independence assumptions were relaxed and the structure of the Bayesian classifier was learned from the data. To make sure that all attributes contribute in classification, class variable has to be a parent for each and every attribute. Formally, the posterior probability *P*(*C*|*A*_1_, …, *A*_*n*_) is required to involve all attributes (Friedman et al., 1997). Therefore, to resolve the risk of missing some vital attributes for classification, here the structure of the classifiers include the structure of the naïve Bayes as a basis. Then, to include the dependencies between attributes, more edges are added to the structure of naïve Bayes classifier. Extracting these additional edges from data is performed following the structure learning algorithms in Bayesian networks. An example of augmented naïve Bayes classifier is shown in Figure 4.

### Decoding-classification

The present study aims to design classifiers to categorize the brain responses associated with stimulus classes, i.e., 10 digit classes. To reach this goal, the response from only one brain lobe (frontal, occipital, parietal, or temporal) was proceed at a time.

Classifiers were trained to recognize cognitive states associated with watching hand written digits using the evoked pattern of functional activity (*t*-statistic). To assess classification performance, trials were partitioned into training and test sets (see cross validation below). To decrease the dimensionality of data, informative features (voxels) were selected from the training set before classification. To ensure an unbiased estimate of classification accuracy, classifiers were trained using the selected features from training set and evaluated on the test set. This is then compared to chance level performance, which in this classification is 10% as there are 10 classes.

### Cross validation

To verify the whole information contained in the dataset, cross validation is frequently used. Therefore, a 10-fold cross validation was applied. In a 10-fold cross validation, all dataset was randomly partitioned to 10-folds with each fold including equal number of samples from all classes. Each time a different fold is left out as the test data and the remaining nine-folds set up the training data. All classification procedure would be repeated 10 times and the mean of the resulted classification accuracies is reported as the final classification accuracy. Various classifiers were applied to the same training sets and evaluated on the same test sets (cross validation folds were the same for all pattern analysis).

It should be noted that all training process used just the training data and the test data did not play any role in feature selection or training classifier.

### Voxel selection

As it is mentioned above, feature selection must be applied just on the training data. Feature selection is employed to choose relevant features (voxels) and remove irrelevant ones (useless features, carry no discriminant information) to improve the generalization capability/performance of the classifiers.

Fisher score, a supervised univariate filter method, was used for voxel selection. A supervised univariate filter method evaluates individual features according to their separability power, independently from other features, and without applying any learning algorithm. Such methods are both computationally efficient and less prone to overfitting than other strategies for feature selection like multivariate methods or wrapper and embedded ones.

Fisher score is designed to optimize sample separability (Zhao et al., 2013). Fisher score chooses features which have the similar values for samples drawn from the same class and different values for samples from different classes (Duda et al., 2001) with the following formulation

(7) $$Fisher\;score\;\left(f_{i}\right)=\frac{\sum_{j\;=\;1}^{c}n_{j}{\left(\mu_{i,j}-\mu_{i}\right)}^{2}}{\sum_{j\;=\;1}^{c}n_{j}\sigma_{i,j}^{2}}.$$

In Equation (7) *c* is the number of classes, μ_*i*_ is the mean of the feature *f*_*i*_, *n*_*j*_ is the number of samples in the *j*th class, and μ_*i, j*_ and $\sigma_{i,j}^{2}$ are the mean and the variance of *f*_*i*_ on class *j*, respectively.

The selected voxels are then given as input to the classifiers.

### Classification

After voxel selection, naïve Bayes and augmented naïve Bayes classifiers from the family of Bayesian network classifiers were used for classification. Learning Bayesian network classifiers includes two stages; structure learning and parameter learning. For the naïve Bayes classifier the structure is defined so learning includes just the parameter estimation and it was done through MLE procedure. For augmented naïve Bayes, at first structural learning is required. To specify the structure, effective connectivies between voxels were extracted by applying the Hill Climbing Greedy Search algorithm with an initial empty graph and the score of Bayesian information criteria. Then, corresponding edges to the obtained brain effective connectivities were added to the structure of naïve Bayes classifier to complete the structure of an augmented naïve Bayes classifier. At the end, MLE was employed for parameter learning. In this way by including brain effective connectivities, the main concern about the naïve Bayes classifiers is addressed; the bias induced by the independence assumption no longer exists.

### Similarity analysis

Various brain regions were used in decoding-classification of hand written digits. Here is the question; which brain regions have similar responses to the stimuli? Or which brain regions were evoked by similar aspects of the stimuli? And what were those aspects? These questions were addressed for Brodmann areas (BAs) of the brain (Brodmann and Garey, 2005; Clark et al., 2010). The reason for choosing Brodmann partitioning of brain is that during the past century clinical findings and neurophysiological studies have shown the agreement between micro structural differences and cortical function specialization for these areas (Zilles and Amunts, 2010).

In this study, responses of active voxels in every BA to each stimulus were averaged resulting in one response vector containing 1000 elements (number of stimuli) for each BA. It should be noticed that active voxels don't cover all BAs. In this step, to visualize BAs similarly responding to the stimuli, a data driven exploratory method (hierarchical agglomerative clustering) was applied on the average BAs' responses for all subjects.

Hierarchical agglomerative clustering assumes that some categorical structure exist, but it does not consider any assumption on grouping of BAs into categories. It tries to discover the categorical divisions of BAs and reveal them in hierarchical cluster trees (Hastie et al., 2009).

## Results

### Decoding-classification

**Figure 5:**
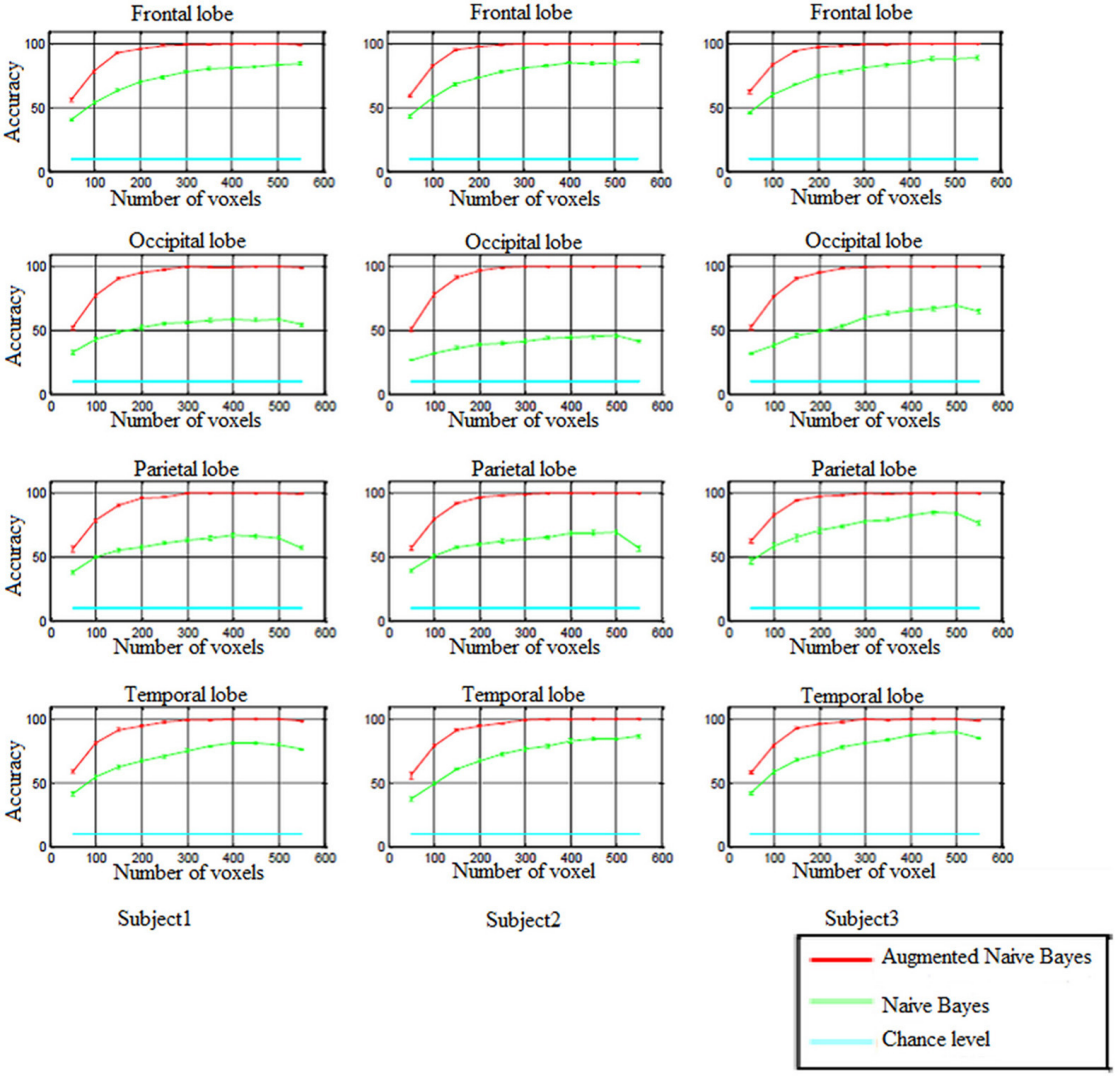
**The accuracy of decoding-classification (mean accuracy ± SEM) vs. number of voxels 50–550 (horizontal axis) used for classification in different lobes for all subjects**.

In order to perform decoding-classification of hand written digits in different brain lobes (frontal, occipital, parietal, and temporal), after partitioning the data to the train and test sets and voxel selection, naïve Bayes and augmented naïve Bayes classifiers were used and classification accuracy was obtained for each cross-validation. Figure 5 shows the accuracy of decoding-classification, mean accuracy ± standard error of mean (SEM), vs. the number of voxels used for classification (50–550) in different lobes for all subjects.

In all brain lobes performance of naïve Bayes and augmented naïve Bayes classifiers were significantly above chance level [Kolmogorov–Smirnov (KS) test with significance level of 0.01]. As it is shown in Figure 5, augmented naïve Bayes classifier produces more accurate decoding (KS test with significance level of 0.01).

When using naïve Bayes classifier, the accuracy of decoding-classification was significantly different in different brain lobes (KS test with significance level of 0.01). However, applying the augmented naïve Bayes classifier, there wasn't a significant difference in the accuracy of decoding-classification for different brain lobes (KS test with significance level of 0.01).

Since selecting approximately 300 voxels for decoding-classification results in accuracy levels of more than 99% (as Figure 5 shows), in the following, the distribution of these selected voxels and effective connectivities among them were investigated in Brodmann areas (BA) of each brain lobe.

Average contribution of voxels from BAs of each brain lobe in 10-fold classifications was studied. Table 1 shows BAs with a contribution of more than 15% in different brain lobes for all subjects.

**Table 1:** **BAs with contribution of more than 15% in different brain lobes for each subject**.

	**Subject1**	**Subject2**	**Subject3**
Frontal lobe	BA6 (28%)	BA6 (29%)	BA6 (26%)
Occipital lobe	BA18 (48%)	BA18 (54%)	BA18 (40%)
	BA19 (31%)	BA19 (27%)	BA19 (28%)
Parietal lobe	BA40 (36%)	BA40 (31%)	BA40 (30%)
	BA7 (31%)	BA7 (29%)	BA7 (30%)
Temporal lobe	BA21 (21%)	BA21 (22%)	BA21 (18%)
	BA22 (17%)	BA22 (18%)	BA22 (18%)

For example the 29% contribution of BA6 in subject 2 means that 29% of the 300 best selected voxels of frontal lobe were from voxels of BA6.

To determine the most important BAs in decoding-classification of hand written digits selected voxels were investigated from another point of view; voxels commonly selected in more than five-folds from all 10-folds (common voxels) were looked for. Common voxels from BAs of each brain lobe were determined. Table 2 shows BAs including more than 15% of common voxels in different brain lobes for all subjects.

**Table 2:** **BAs including more than 15% of common voxels in different brain lobes for all subjects**.

	**Subject1**	**Subject2**	**Subject3**
Frontal lobe	BA6 (30%)	BA6 (29%)	BA6 (24%)
Occipital lobe	BA18 (47%)	BA18 (49%)	BA18 (43%)
	BA19 (31%)	BA19 (30%)	BA19 (27%)
Parietal lobe	BA40 (36%)	BA40 (32%)	BA40 (31%)
	BA7 (31%)	BA7 (26%)	BA7 (29%)
Temporal lobe	BA22 (19%)	BA22 (19%)	BA22 (20%)

For example the contribution of 30% for BA6 of subject 1 means that 30% of all common voxels in frontal lobe were from voxels of BA6.

**Figure 6:**
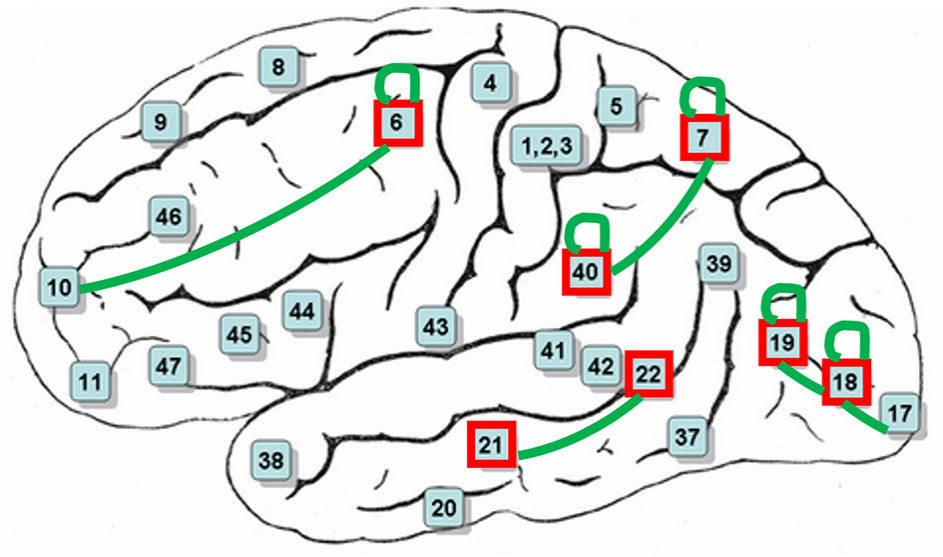
**BAs mostly contributed in decoding-classification of hand written digits (red boxes) and common connectivities among all subjects (green connections)**.

After decoding-classification, brain connectivities were investigated to determine highly connected BAs in the visual experiment of watching hand written digits. Connectivities with a frequency of more than 5% of their total population are reported in Table 3. It is clear that these frequent connectivities are mostly common among all subjects in current experiment (Figure 6).

**Table 3:** **Connectivities with a frequency of more than 5% from the total connectivities (common connectivities among all subjects are printed in bold)**.

	**Subjects1**	**Subjects2**	**Subjects3**
Frontal lobe	**{BA6,BA6}** 10%	**{BA6,BA6}** 9%	**{BA6,BA6}** 7%
	{BA6,BA9} 7%	{BA6,BA9} 9%	**{BA6,BA10}** 6%
	**{BA6,BA10}** 7%	**{BA6,BA10}** 7%	
	{BA6,BA8} 6%	{BA9,BA10} 6%	
		{BA6,BA8} 5%	
Occipital lobe	**{BA18,BA19}** 27%	**{BA18,BA18}** 36%	**{BA18,BA19}** 19%
	**{BA18,BA18}** 24%	**{BA18,BA19}** 24%	**{BA18,BA18}** 16%
	**{BA19,BA19}** 11%	**{BA17,BA18}** 10%	**{BA17,BA18}** 16%
	**{BA17,BA18}** 11%	**{BA19,BA19}** 8%	{BA17,BA17} 12%
	{BA17,BA19} 6%		{BA17,BA19} 10%
			**{BA19,BA19}** 8%
Parietal lobe	**{BA7,BA40}** 19%	**{BA7,BA40}** 16%	**{BA7,BA40}** 16%
	**{BA40,BA40}** 15%	**{BA40,BA40}**12%	**{BA40,BA40}** 11%
	**{BA7,BA7}** 14%	**{BA7,BA7}** 12%	**{BA7,BA7}** 11%
	{BA3,BA40} 5%		{BA3,BA40} 6%
			{BA3,BA7} 6%
Temporal lobe	{BA20,BA21} 7%	**{BA21,BA22}** 7%	**{BA21,BA22}** 6%
	**{BA21,BA22}** 6%	{BA21,BA21}5%	
	{BA21,BA21} 5%	{BA20,BA39}5%	
	{BA20,BA22} 5%		

For example a frequency of 7% for connectivity between BA6 and BA10 of subject 1 means that 7% of all extracted connectivities in frontal lobe were between voxels of BA6 and BA10.

**Figure 7:**
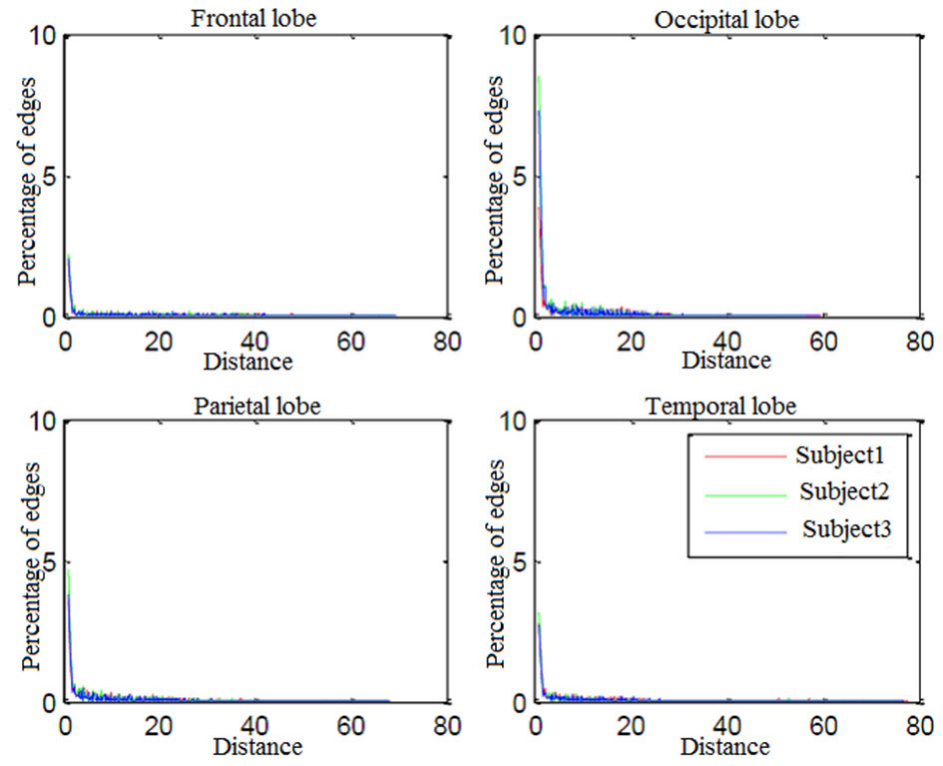
**Percentage of edges with Euclidean distance of 0−80 between endpoints**.

Looking more closely at Table 3, it can be seen that the frequency of connectivities within BAs (connectivities with both endpoints in the same BA) is considerable. This impelled us to investigate distribution of the distances between endpoints of connectivities. Figure 7 shows the edge percentages with Euclidean distance of 0–80 between endpoints. As Figure 7 shows percentages of edges with shorter distance is higher in all brain lobes, particularly occipital and parietal.

### Similarity analysis

**Figure 8:**
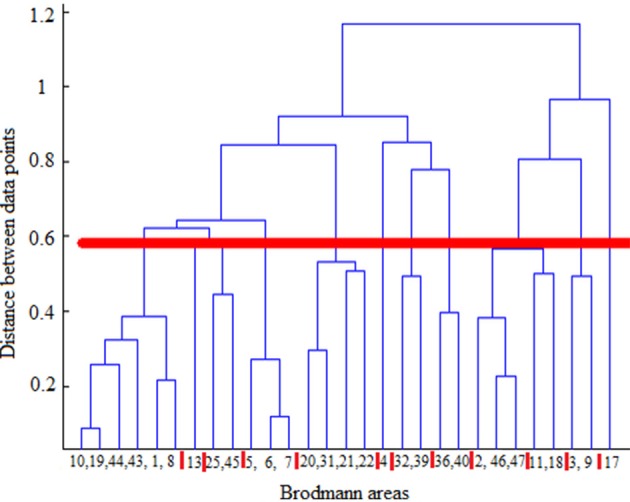
**Cluster trees using the correlation distance metric and furthest distance algorithm for computing the clusters' distances**. Red horizontal line depicts the half of the maximum distance between data points and red vertical lines between leaves separate clusters. From left to right clusters are: (10,19,44,43,1,8), (13), (25,45), (5,6,7), (20,31,21,22), (4), (32,39), (36,40), (2,46,47), (11,18), (3,9), (17).

**Figure 9:**
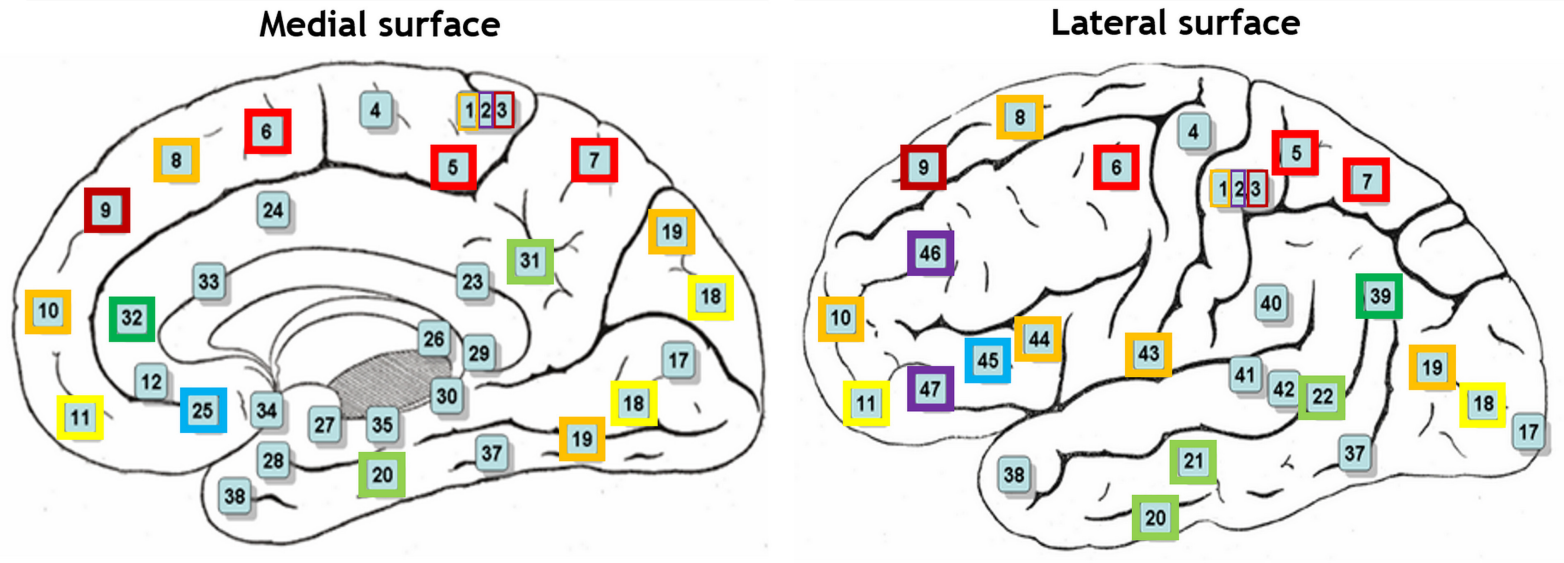
**BAs on lateral and medial surfaces of brain**. BAs similarly responding to the stimuli are in the same color.

Hierarchical agglomerative clustering was applied on the average BAs' responses for all subjects to discover the similarities in responding to the stimuli. The distance matrix for BAs' responses employing the correlation distance metric was computed and Figure 8 shows the cluster trees using the same distance metric to apply furthest distance algorithm for computing the distance between clusters. Considering the largest clusters with a distance between data points less than half of the maximum distance between data points (12 clusters), some BAs are grouped: (BA17), (BA3, BA9), (BA2, BA46, BA47), (BA11, BA18), (BA36, BA40), (BA32, BA39), (BA4), (BA13), (BA25, BA45), (BA5, BA6, BA7), (BA31, BA20, BA21, BA22), (BA10, BA19, BA44, BA43, BA1, BA8). Figure 9 shows BAs present on lateral and medial surfaces of brain with similar responses to the stimuli in the same color.

## Discussion and conclusions

Although the experiment was only a visual task, in all brain lobes performance of naïve Bayes and augmented naïve Bayes classifiers was significantly above chance level. It seems that the information of different brain lobes may be used to successfully accomplish the decoding-classification of hand written digits due to the fact that their activity may be associated with different features of stimulus images including superficial or semantic features.

Among all classifiers augmented naïve Bayes classifier (which considers the brain effective connectivity) produces more accurate decoding. Brain connectivities were extracted following the structure learning methods in Bayesian networks. Employing connectivity information in decoding-classification of hand written digits was greatly advantageous and this approach is most likely to open a way to solve the decoding problem. This advantage of augmented naïve Bayes or the effect of employing effective brain connectivities is greater in occipital and parietal lobes, so decoding-classification is more dependent on effective brain connectivities in those brain lobes.

When using naïve Bayes classifier, the accuracy of decoding-classification was significantly different in different brain lobes and this would be interpreted as lower capability of some brain lobes in comparison to others in decoding-classification of hand written digits. However, applying the augmented naïve Bayes classifier, a more realistic model including the effective connectivities, there wasn't a significant difference in the accuracy of decoding-classification for different brain lobes. Therefore, all brain lobes were successful in decoding-classification of hand written digits while they might have different mechanisms for this classification (for example using low/high level features).

One might think the improvement in results using augmented naïve Bayes (which takes the connectivity into account) is only due to the increasing complexity of the classifier. To examine this conjecture, by increasing the number of voxels, naïve Bayes classifier was used and the results indicated overfitting. Therefore, the more accurate decoding-classification of augmented naïve Bayes is not due to the addition of complexity level, but utilization of connectivity information raises the accuracy significantly.

Functions of the most contributing BAs in decoding-classification of hand written digits (Figure 6) related to the performed visual experiment are listed in Table 4 (Luria, 1976; Mesulam, 2000; Brodmann and Garey, 2005; Clark et al., 2010; Trans Cranial Technologies Ltd., 2012). As Table 4 shows these BAs are well-known areas in both language and semantic processing which is in line with our expectations about active BAs in watching hand written digits. Since digits are part of the language aspects and besides, there were classes of hand written digits with considerable variety in their visual feature while sharing the same semantic aspects.

**Table 4:** **Functions of mostly active BAs related to the fMRI visual experiment/task**.

Frontal lobe	BA6: Response to visual presentation of letters and pseudo-letters (left) Language processing
Occipital lobe	BA18: detection of patterns, word encoding, response to visual word form (left)
	BA19: detection of patterns, word encoding
Parietal lobe	BA7: language processing, Semantic categorization tasks
	BA40: language processes, semantic processing, writing of single letters
Temporal lobe	BA21: semantic processing (left)
	BA22: receptive language, Semantic processing (left)

Percentages of edges with shorter distance is higher in all brain lobes particularly occipital and parietal. Since these two were brain lobes for which decoding-classification was mostly improved by employing connectivity information, we conclude that connectivities with short distances between the endpoints are more efficient in decoding-classification and local brain networks might be responsible for this classification of hand written digits in brain.

This is the first study on the decoding-classification of this stimulus set, although hand written digits are among the most frequent stimuli in today's modern life and present research successfully performed decoding-classification of hand written digits 0–9. This success is due to the number of samples, voxel selection approach, number of chosen voxels and characteristics of applied classifiers in current research in comparison to previous studies. We used 100 samples for each hand written digit, Fisher score for voxel selection, 50–550 chosen voxels and naïve Bayes or augmented naïve Bayes classifiers.

The closest previous studies are Eger et al. (2009), van Gerven et al. (2010), and somehow Damarla and Just (2013).

Eger et al. (2009) used eight samples for each stimulus—computer (not hand written) digits 2, 4, 6, and 8 or images of 2 dots, 4 dots, 6 dots, and 8 dots, chose 1000 voxels most significantly activated across all sample stimulus conditions regardless of their notation (digits or dots), used linear SVM classifier and gained a discrimination accuracy <60% in decoding-classification of digits. It is clear that number of samples in Eger et al. (2009) is much less than the number of samples in current study (8 vs. 100). Eger et al. (2009) chose 1000 voxels, but there is a drawback with their voxel selection approach; voxels were chosen regardless of their notation while comparing the patterns evoked by computer digits and dots, the format was accurately discriminated with ~80%. It seems that to improve the decoding-classification, stimulus formats should be considered in voxel selection approach. Another weak point of Eger et al. (2009) in comparison to present research is their classifier, linear SVM. Linear SVM looks for linear separators while Bayesian classifiers are non-linear ones and present probabilistic models of the data instead of separators. It should be mentioned that at first, decoding-classification for each digit pair was investigated and application of linear SVM derived results in accordance with Eger et al. (2009) while results of other non-linear classifiers were significantly better, hence application of multiple-class classifiers seemed possible.

van Gerven et al. (2010) used 50 samples for each hand written digits 6 and 9, used Bayesian multivariate analysis with sparsifying spatio-temporal prior and obtained a discrimination accuracy more than 70%. In current study there were 100 samples for each hand written digit, this is twice the number of samples in van Gerven et al. (2010). Besides, the decoding-classification procedure of present research was applied on the data set of van Gerven et al. (2010) and just using the information of tens of voxels in naïve Bayes or augmented naïve Bayes classifiers, classification accuracies more than 90% were obtained.

Damarla and Just (2013) used 16 samples for each quantity 1, 3, and 5 in two modes (digit-picture and picture), chose 120 voxels whose vector of response intensity to the set of stimulus items were the most stable, applied naïve Bayes classifier and gained a discrimination accuracy of 66% in decoding-classification in digit-picture mode. On one side, number of samples in Damarla and Just (2013) is much less than the number of samples in current study (16 vs. 100). One the other side, Damarla and Just (2013) used 120 most stable voxels in responding to stimulus set regardless of the stimulus mode; picture, or digit-picture. It seems that both increasing the number of voxels and alternative voxel selection approaches (considering the stimulus mode) could improve their classification accuracy.

The same decoding-classification approach was applied one more time just with altering the classifier to linear SVM this time. Parameters of linear SVM were optimized on the training sets. Performance of linear SVM was almost always at chance level except occipital lobe where classification accuracy was somehow above chance level. This could be due to this fact that linear SVM looks for linear separators while Bayesian classifiers are non-linear ones and present probabilistic models of the data instead of separators. The main reason for the popularity of linear SVM in neuroscience studies is due to the usual shortage of samples available. Non-linear SVM or probabilistic version of SVM (relevance vector machine) might result in better decoding-classification of present data set.

To increase the confidence in the results some regions were used as control regions to re-run the analysis. Brodmann area 2 (BA2) was one of the control regions. It is a primary somatosensory area that localizes touch, temperature, vibration, and pain. BA2 is also responsible for movement organization and voluntary hand/tongue movement (Luria, 1976; Mesulam, 2000; Brodmann and Garey, 2005; Clark et al., 2010; Trans Cranial Technologies Ltd., 2012). Therefore, there is no expectation to have the capability of decoding-classification and there is no significant, both anatomical and functional connections between BA2 and some areas that had high responses. The decoding-classification procedure of current study was applied on BA2, however this attempt was not successful and resulted in accuracies not significantly different (*p* = 0.95) from the chance level (*p* < 0.05).

It was preferred to use a greedy search approach to estimate the structure of the Bayesian Network compared to Tree Augmented Naive Bayes due to following reasons:

1. The major motivation for the application of tree augmented naïve Bayes is the computational efficiency, not the better performance (Friedman et al., 1997).
2. The brain is a highly complex set of connected functional regions. Therefore, a simple tree-structure is not expected to be a perfect model of connectivities covering its detail (Smith et al., 2009).
3. Since we were interested in studying the distribution of connections, it was not acceptable to impose extra condition of having a tree-structured network.

Another outstanding characteristic of the current research is investigating the whole brain, rarely done in previous decoding studies. This brought us the opportunity of determining efficient areas of different brain lobes for decoding-classification of hand written digits and comparing the capability of brain lobes in this experiment. The experiment included watching hand written digits. According to the literature, if just the visual aspect of stimuli was considered, occipital lobe would have been used; if the quantity aspect of stimuli was the focus, parietal lobe would have been applied and if only the language aspect of stimuli was considered, frontal and temporal lobe would have been used. Since the paradigm of the experiment didn't try to focus on either aspects, it was not clear that for the task of watching hand written digits which aspect is the most efficient in decoding-classification. Therefore, digits were classified using different lobes and they were compared in regard of classification accuracy.

Different mechanisms and functions for BAs were evidenced so far, however various BAs were actively participating in decoding-classification of hand written digits. To investigate this observation, BAs similarly responding to each stimulus were determined. Hierarchical agglomerative clustering was applied on the average BAs' responses for all subjects to discover the similarities.

According to the literature, BAs 17–19 are active in visual processing, working memory, and language (response to visual word forms; Trans Cranial Technologies Ltd., 2012). BA17, BA18, and BA19, all three deal with visual processing, BA17 is considered as primary or projection visual cortex, while BAs 18 and 19 are secondary or association cortex. They were not grouped together, and they were grouped in three different clusters; (BA17), (BA11, BA18), (BA10, BA19, BA44, BA43, BA1, BA8). It can be seen that BA17 built a cluster individually, while BA18 and BA19 shared their clusters with areas not active in visual processing. It could be understood that they followed different mechanisms in responding to the same stimulus set and they responded to different aspects of them. Based on this observation, it looked like that visual processing was the dominant mechanism just in BA17 and not the other two areas during the experiment of watching hand written digits.

BA11 was in the same group with BA18. Besides, BA11 is a part of dorsolateral prefrontal cortex (DL-PFC) involved in motor tasks (planning, organization, or regulation) and working memory (working memory is involved in diversity of cognitive process including language; Luria, 1976; Mesulam, 2000; Brodmann and Garey, 2005; Clark et al., 2010; Trans Cranial Technologies Ltd., 2012). Therefore, according to the recognized functions of BA18, it seems that working memory was the main task of these both areas.

BA19 was not in the same group with BA17 and BA18 which were recognized to be responsible for visual processing and working memory, respectively. Therefore, it looked like that BA19 deals mostly with language aspects of the experiment. Moreover, language processing is a common function of all other BAs sharing the same group with BA19 i.e., BA8, BA10, BA43–44.

BA2 and BA3 are responsible for movement organization and voluntary hand/tongue movement (Luria, 1976; Mesulam, 2000; Brodmann and Garey, 2005; Clark et al., 2010; Trans Cranial Technologies Ltd., 2012) and BA9, BA46, and BA47 are parts of dorsolateral prefrontal cortex (DL-PFC), as mentioned above, involved in motor tasks and working memory. Therefore, having the clusters (BA3, BA9), (BA2, BA46, BA47) shows that motor organization was their dominant activity.

BA31, BA20, BA21, and BA22 also built a cluster and language processing is the recognized function of all these areas (Luria, 1976; Mesulam, 2000; Brodmann and Garey, 2005; Clark et al., 2010; Trans Cranial Technologies Ltd., 2012). Hence, they were commonly active in language processing in this experiment. Moreover, according to BAs' functions in literature, (BA36, BA40), (BA32, BA39) were two more clusters with identified mechanism of working memory and language processing respectively, (Trans Cranial Technologies Ltd., 2012).

Therefore, during our experiment of watching hand written digits there were active networks; visual, working memory, motor and language processing, and performing voxel selection, language processing areas were chosen to participate in decoding-classification not the motor processing ones.

The results of decoding-classification (the proposed classifiers) could be efficiently employed in brain–computer interfaces. Besides, the results of decoding-classification and similarity analysis may assist us to detect malfunction areas or mechanisms and consider them in proposing brain–computer interfaces or therapeutic procedures. As a case in point, in designing neuro-feedback systems, it determines the mechanism to be improved. Also, in rehabilitation procedures, transcranial magnetic stimulation could be applied to areas with similar activity to gain more and faster improvement.

It is noteworthy that there were no assumptions on the stimulus sets or the experiment conditions. Hence, there won't be any limitations in generalizing applied approach to other decoding-classification tasks. In this way, satisfactory results of present study may promise success in separating representations of other challenging stimulus classes.

To improve the quality and precision of obtained results, including more subjects will be useful. Another suggestion is to employ structural connectivity along with effective connectivity information. Decoding-classification of other stimulus families, using connectivity informed classifiers will also be the direction of further studies.

## Author contributions

EY is a Ph.D. student who introduced a new method for brain decoding (classification). She took into account the connectivity for brain decoding along with the application of Bayesian networks in order to do the decoding classification of hand written digits. GH is the supervisor of Miss EY's Ph.D. thesis, who directed the student in the field of brain decoding and helped her to adapt the new methods for fMRI analysis and provided the opportunity for her to acquire fMRI data and to present a scientifically sound research in terms of validity, evaluation etc.

### Conflict of interest statement

The authors declare that the research was conducted in the absence of any commercial or financial relationships that could be construed as a potential conflict of interest.
